# PReS-FINAL-2036: Proteins of the lectin pathway of the complement system in juvenile idiopathic arthritis

**DOI:** 10.1186/1546-0096-11-S2-P49

**Published:** 2013-12-05

**Authors:** C Petri, S Thiel, JC Jensenius, B Kuno-Møller, T Herlin

**Affiliations:** 1 Department of Pediatrics, Aarhus Univerisity Hospital, Aarhus, Denmark; 2 Department of Biomedicine, Aarhus University, Aarhus, Denmark; 3 Department of Clinical Immunology, Aarhus Univerisity Hospital, Aarhus, Denmark

## Introduction

The complement system plays a crucial role in the pathogenesis of various inflammatory processes. The lectin pathway of the complement is activated through the recognition of pathogens or altered self-structures by the pattern recognition molecules (PRMs) mannan-binding lectin (MBL), H-ficolin, L-ficolin and M-ficolin in collaboration with MBL-associated serine proteases (MASPs). PRMs reportedly play a role in rheumatoid arthritis (RA), and a recent study indicated a correlation between the concentration of these proteins and RA disease activity. Knowledge regarding the role of lectin pathway proteins in juvenile idiopathic arthritis (JIA) is lacking.

## Objectives

The aim of the study was to evaluate the possible pathogenic role of the PRMs like MBL, H-ficolin and M-ficolin, MASP-1,-2,-3 as well as the two alternative splice products, MAp19 and MAp44 of the genes encoding the MASPs. We tested paired samples of plasma and synovial fluid (SF) from patients with oligoarticular and systemic JIA.

## Methods

We measured MBL, M-ficolin, H-ficolin, MASP-3, MASP-2, MASP-1, MAp44 and MAp19 in plasma/serum and synovial fluid (SF) of 136 children with oligoarticular JIA (persistent subtype) and 28 children with systemic JIA. The concentrations of the nine proteins were measured by in-house time-resolved immunoflurometric assays (TRIFMA) using monoclonal antibodies. The principle of this assay is the same as for sandwich ELISA, only fluorescence rather than enzyme activity is used for the read-out. In brief, microtitre wells are coated with monoclonal antibody, incubated with dilutions of the test samples, then with biotinylated monoclonal antibody, and finally developed with europium-labeled streptavidin. The concentrations of the analytes are read from parallelly constructed standard curves.

## Results

Concentrations of MASP-3, MASP-2, M-ficolin and MASP-1, respectively between the oligoarticular and the systemic subtype differed significantly in plasma. Measurements of serum samples showed significant difference for M-ficolin, H-ficolin, MAp44 and MASP-1 between the two groups.

SF/plasma ratio of the lectin pathway proteins were calculated in paired samples for oligoarticular JIA (n = 36) and the systemic onset JIA (n = 11). We observed significant higher levels in plasma than in SF for both subtypes with P values <0.01, while the significance was lower for H-ficolin (p = 0.04) and MBL (p = 0.1549) in the systemic group. Conversely, for MASP-3 we observed significantly higher concentration in SF than in plasma - ratio 1.97 (1.61; 2.32) (p < 0.001) for the oligoarticular subtype and 2.59 (1.73;3.44)(p = 0.0033) for systemic subtype.

## Conclusion

The level of the proteins is higher in plasma than in SF for both oligoarticular JIA and systemic JIA, except for MASP-3, indicating that MASP-3 somehow is secreted into or produced locally in the joint. Further investigation and quantification of the correlation between the concentrations and inflammatory markers is in progress.

## Disclosure of interest

None declared.

